# Single-cell RNA sequencing uncovers cellular heterogeneity and the progression of heterotopic ossification of the elbow

**DOI:** 10.3389/fphar.2024.1434146

**Published:** 2024-07-08

**Authors:** Chi Zhang, Dan Xiao, Li Shu, Maoqi Gong, Xinghua Liu, Xieyuan Jiang

**Affiliations:** ^1^ Department of Orthopedics, Peking University International Hospital, Beijing, China; ^2^ Biomedical Engineering Department, Institute of Advanced Clinical Medicine, Peking University, Beijing, China; ^3^ Department of Orthopedic Surgery, Beijing Jishuitan Hospital, Beijing, China; ^4^ Department of Orthopedic Surgery, Beijing Chaoyang Hospital, Capital Medical University, Beijing, China

**Keywords:** heterotopic ossification of the elbow, single-cell RNA sequencing, bone formation, endochondral ossification, heterogeneity, osterix

## Abstract

Heterotopic ossification of the elbow (HOE) is a complicated pathologic process characterized by extra bone formation in the elbow. Bone formation is a complex developmental process involving the differentiation of mesenchymal stem cells into osteoblasts. The aim of this study was to explore the cellular origin and progression of HOE by single-cell RNA sequencing. We identified 13 clusters of cells in HOE and further analyzed the subclusters for 4 of the main cell types. Six subclusters of osteoblasts, nine subclusters of chondrocytes, six subclusters of fibroblasts, and five subclusters of mononuclear phagocytes (MPs) were identified and analyzed. The new findings on osterix (OSX) and SOX9 expression in osteoblast subclusters and chondrocyte subclusters indicate that HOE is mediated through endochondral ossification. Further identification of the corresponding signature gene sets of distinct subclusters indicated that subclusters of osteoblasts_3, osteoblasts_4, osteoblasts_5, and osteoblasts_6 are relatively more mature during the osteoblastic progression of HOE. The trajectory analysis of the osteoblasts demonstrated that some genes were gradually downregulated, such as CRYAB, CCL3, SFRP4, WIF1, and IGFBP3, while other critical genes were upregulated, such as VCAN, IGFBP4, FSTL1, POSTN, MDK, THBS2, and ALPL, suggesting that these factors may participate in HOE progression. Cell–cell communication networks revealed extensive molecular interactions among the 13 HOE clusters. Ligand–receptor pairs for IL6, COL24A1, COL22A1, VWF, FZD6, FGF2, and NOTCH1 were identified, suggesting that multiple signaling pathways may be involved in HOE. In conclusion, this study provided the cellular atlas for HOE. We have established a greater extent of the heterogeneity of HOE cells than previously known through transcriptomic analysis at the single-cell level. We have observed gradual patterns of signature gene expression during the differentiation and maturation progression of osteoblasts from stem cells in HOE with higher resolution. The cell heterogeneity of HOE deserves further investigation to pave the way for identification of potential targets for HOE early diagnosis and therapeutic treatment.

## Introduction

Heterotopic ossification (HO) is a complicated pathologic process characterized by the formation of extra bone in soft tissues, such as muscles, peri-articulations, ligaments, and tendons. It is known as a complication of trauma, surgery, blast, spinal cord injury, and other stress damages (Shimono et al., 2011; Regard et al., 2013; Ranganathan et al., 2015). HO clinical features include limited motion range around the involved joint; bony ankylosis in severe cases; deformity in the cervical spine, elbow, shoulder, and fingers, jaw exostosis; and temporomandibular joint ankylosis (Zhao et al., 2020).

Compared with the other joints, the elbow commonly shows HO development. Heterotopic ossification of the elbow (HOE) is usually localized and sometimes has a self-limited course without long-term symptoms, and its incidence may be underreported (Summerfield et al., 1997). Although HO-associated osteosis is histologically similar to the lamellar structure of the bone, it is more active metabolically and does not come with a true periosteal layer (Jupiter et al., 2003). Once HO begins to develop, there are no options of treatment available to prevent or revert the HO process. Clinical presentations of HO include pain, soft tissue swelling, tenderness, higher temperature, and progressive loss in the joint motion range. These postoperative symptoms are mistaken for an early infection. By contrast, extensive HOE can lead to clinically important contractures and even complete ankylosis (Baldwin et al., 2011), which can impede performing daily living activities, especially when the dominant extremity is affected. HOE-induced complete ankylosis can be debilitating to patients and becomes a therapeutic challenge to the treating surgeon (Garland, 1998). Surgical treatment for ankylosis of the elbow is both risky and difficult. To date, the mechanism of HOE progression is not clear.

New bone is produced by a process called ossification. Bone formation includes two models of endochondral ossification and intramembranous ossification. Endochondral ossification forms a bone through a cartilage intermediate, while intramembranous ossification directly forms the bone on the mesenchyme. In general, endochondral ossification is involved in long bone formation, while intramembranous ossification is involved in flat bone formation. Bone formation is a complex developmental process involving osteoblast differentiation from mesenchymal stem cells (MSCs) (Zhang, 2012). Osteoblast differentiation from stem cells is mediated by some important transcription factors, including IHH, RUNX2, and Osterix (OSX, also named SP7) (Zhang, 2012). OSX, as the only osteoblast-specific transcription factor identified so far, is required for bone formation and osteoblast differentiation (Nakashima et al., 2002; Zhang et al., 2008). OSX was originally discovered as a bone morphogenetic protein-2 (BMP2) inducible gene in mesenchymal stem cells, and OSX knockout mice lack bone formation completely (Nakashima et al., 2002). On the other hand, cartilage formation starts with mesenchyme cell condensation, followed by their differentiation into chondrocytes. SOX9 is a high-mobility-group domain transcription factor that is essential for chondrocyte differentiation and cartilage formation (Bi et al., 1999). No cartilage was developed from SOX9 knock out embryonic stem cells.

We still do not well understand the cellular origin, pathogenesis, and underlying mechanisms of HOE. It is critical to analyze cell subsets, which might shed light on HOE progression. A better understanding of its mechanisms may help develop potential new strategies for early diagnosis and therapeutic treatment. In this study, we performed single-cell RNA sequencing to elucidate the cellular heterogeneity in HOE and to reveal HOE progression.

## Materials and methods

### Patient samples

HOE patients were recruited from Beijing Research Institute of Traumatology and Orthopedics, Beijing Jishuitan Hospital. This work was approved by the ethics committee of Beijing Jishuitan Hospital and complied with principles of the Declaration of Helsinki (#202009-16). Written informed consent was obtained from all participants involved. We obtained tissue samples from patients during the operation.

### Tissue dissociation and preparation

Fresh HOE tissues were kept in the sCelLive™ Tissue Preservation Solution (Singleron) on ice after surgery within 30 min. The specimens were washed three times with Hanks’ Balanced Salt Solution, minced into small pieces, and then digested with 3 mL sCelLive™ Tissue Dissociation Solution using a Singleron PythoN™ Tissue Dissociation System at 37°C for 15 min. The cell suspension was collected and filtered through a 40-µm sterile strainer. The GEXSCOPE^®^ red blood cell lysis buffer (RCLB) was added, and the mixture (cell:RCLB = 1:2) was incubated for 5–8 min at room temperature to remove the red blood cells. The mixture was then centrifuged at 300 ×g at 4°C for 5 min to remove the supernatant and suspended with PBS/DMEM. The samples were stained with Trypan blue, and the cell viability was determined microscopically. Cells from three patients were mixed for further analysis.

### RT and amplification and library construction

Single-cell suspensions (2×10^5^ cells/mL) with PBS were loaded onto a microwell chip using the Singleron Matrix^®^ single-cell processing system. Barcoding beads were collected from the microwell chip, followed by reverse transcription of the mRNA captured by the barcoding beads. cDNA was obtained. The amplified cDNA was fragmented and ligated with sequencing adapters. The single-cell RNA sequencing libraries were generated based on the protocol of the GEXSCOPE^®^ Single-Cell RNA Library Kits (Singleron) (Dura et al., 2019). Individual libraries were diluted to 4 nM, pooled, and sequenced on Illumina NovaSeq 6000 with 150-bp paired-end reads.

### Primary analysis of raw read data

Raw reads from single-cell RNA sequencing were processed to obtain gene expression matrixes using the CeleScope (https://github.com/singleron-RD/CeleScope) v1.9.0 pipeline. Briefly, raw reads were processed using CeleScope to remove low-quality reads using Cutadapt v1.17 to trim poly-A tail and adapter sequences. The cell barcode and unique molecular index (UMI) were then extracted. STAR v2.6.1a was used to map reads to the reference genome GRCh38 (Ensembl version 92 annotation), as previously described (Dobin et al., 2013). UMI counts and gene counts of each cell were obtained using featureCounts v2.0.1 software and used to obtain expression matrix files for further analysis (Liao et al., 2014).

### Quality control, dimension reduction, and clustering

The cells were filtered by gene counts below 200 and the top 2% gene counts and the top 2% UMI counts. Cells with over 26% mitochondrial content were removed. After filtering, 14,925 cells were retained for the following analyses. Functions from Seurat v3.1.2 were used for dimension reduction and clustering (Satija et al., 2015; Stuart et al., 2019). NormalizeData and ScaleData functions were used to normalize and scale expression of all genes. The top 2,000 variable genes obtained using the FindVariableFeautres function were selected for PCA. Using the top 20 principal components, the cells were separated into multiple clusters using FindClusters. The uniform manifold approximation and projection (UMAP) algorithm was applied to visualize cells in a two-dimensional space.

### Statistics and repeatability

The gene expression or gene signature of two groups of cells was compared using unpaired two-tailed Student’s *t*-test. All statistical analyses and presentations were carried out using R. Statistical significance was set at *p* < 0.05.

### Differentially expressed gene analysis

To identify differentially expressed genes (DEGs), the Seurat FindMarkers function was used based on the Wilcox likelihood-ratio test with default parameters, and the genes expressed in more than 10% of the cells in a cluster and with an average log(Fold Change) value greater than 0.25 were selected as DEGs. To address the cell type annotation of each cluster, we combined the canonical marker expression found in the DEGs with knowledge from studies and displayed the marker expression of each cell type with heatmaps/dot plots/violin plots that were generated using the Seurat DoHeatmap/DotPlot/Vlnplot function. Doublet cells were identified as expressing markers for different cell types and were removed.

### Cell type annotation

The cell type identity of each cluster was determined with the canonical marker expression found in the DEGs using the SynEcoSys database. Heatmaps/dot plots/violin plots displaying the marker expression for each cell type were generated using the Seurat v3.1.2 DoHeatmap/DotPlot/Vlnplot function, respectively.

### Pathway enrichment analysis

To explore the potential functions of DEGs, the Gene Ontology (GO) and Kyoto Encyclopedia of Genes and Genomes (KEGG) analyses were conducted using the “clusterProfiler” R package 4.0.1 (Wu et al., 2021). Pathways with a p_adj value less than 0.05 were considered significantly enriched. Gene Ontology gene sets including molecular function (MF), biological process (BP), and cellular component (CC) categories were used as the reference. For Gene Set Variation Analysis (GSVA) pathway enrichment analysis, we used the average gene expression of each cell type as input data using the GSVA package (Hanzelmann et al., 2013).

### Trajectory analysis

Monocle 2 was used to reconstruct the cell differentiation trajectory, as previously described (Qiu et al., 2017). DEGs were used to sort cells in the order of spatial–temporal differentiation. DDRTree was used to perform FindVairableFeatures and dimension reduction. The trajectory was visualized using the plot_cell_trajectory function. CytoTRACE (a computational method that predicts the differentiation state of cells from single-cell RNA sequencing data using gene counts and expression) was used to predict the differentiation potential of monocyte subpopulations, as previously described (Gulati et al., 2020).

### Cell–cell interaction analysis

The cell–cell interaction analysis was performed using CellPhoneDB v2.1.0 based on known receptor–ligand interactions between two cell types/subtypes (Efremova et al., 2020). Cluster labels of all cells were randomly permuted 1,000 times so as to determine the null distribution of average ligand–receptor expression levels of the interacting clusters. Individual ligand–receptor expression was thresholded with a cutoff value based on the average log gene expression distribution for all genes across the cell types. The significant cell–cell interactions were defined as *p*-value <0.05 and average log expression >0.1, and these were visualized using the circlize v0.4.10 R package.

### Transcription factor regulatory network analysis

A transcription factor network was generated by pySCENIC v0.11.0 using the scRNA expression matrix and transcription factors in AnimalTFDB (Van de Sande et al., 2020). GRNBoost2 predicted a regulatory network according to the co-expression of regulators and targets. CisTarget was further applied to exclude indirect targets and explore transcription factor-binding motifs. AUCell was applied for regulon activity quantification for every cell. The top transcription factor regulons with a high regulon specificity score (RSS) were visualized using a pheatmap in R.

## Results

### Cellular constitution of heterotopic ossification of the elbow

**FIGURE 1:**
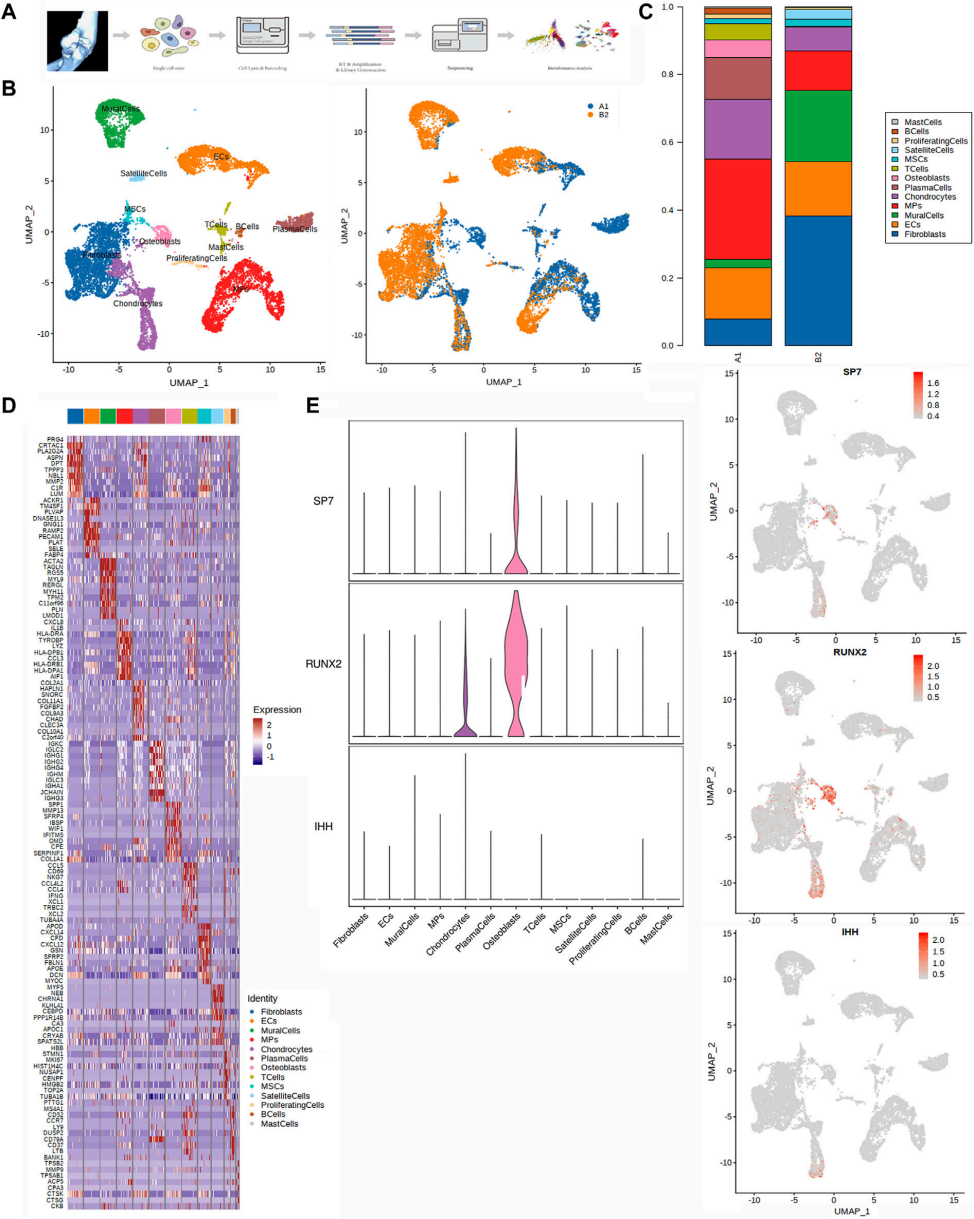
Single-cell transcriptomic analysis of HOE tissues. **(A)** Graphical view of this study roadmap. Single-cell suspensions were obtained from HOE patients, followed by single-cell RNA sequencing. A total of 14,925 qualified single cells were recovered. **(B)** Uniform manifold approximation and projection (UMAP) analysis plot of the 13 identified main cell types in HOE tissues. Left panel colored by cell clusters; right panel colored by tissue samples. A1: HOE samples; B2: control samples. **(C)** Proportion of the main cell types in HOE tissues compared with the control group. **(D)** Heatmap of the top 10 differentially expressed gene sets of these 13 cell clusters. **(E)** Expression profiles of three osteoblast representative genes SP7, RUNX2, and IHH in the cell populations. The left panel shows violin plots showing the normalized expression levels of the three osteoblast representative marker genes across the 13 clusters. The right panel shows the UMAP plot showing the three osteoblast representative canonical marker genes across the 13 clusters.

We performed single-cell RNA sequencing analysis on heterotopic ossification samples from three HOE patients to explore their cellular composition (Figure 1). Adjacent tissues around heterotopic ossification sites were used as a control. Due to the difficulty of obtaining enough cells from the hard tissue of HOE, cells from three HOE patient tissues were mixed together for further analysis. After initial quality control assessment and doublet removal, we obtained single-cell transcriptomes from a total of 14,925 cells, i.e., 6,526 cells from HOE tissues and 8,399 cells from control HOE adjacent tissues. The number of median detected UMIs for the HOE group was 7,045 per cell, with the median detected genes of 1,894 per cell. The number of median detected UMIs for the control group was 7,715 per cell, with the median detected genes of 2,241 per cell.

We performed dimensionality reduction analysis during integrate annotation. Unbiased clustering of the cells identified 13 clusters based on UMAP analyses according to their gene profiles and canonical markers (Figure 1B). A1 represented HOE samples, while B2 represented control samples. The 13 clusters were as follows: 1) mast cells highly expressing TPSAB1, TPSB2, and CPA3; 2) B cells highly expressing MS4A1, CD79A, and CD79B; 3) proliferating cells highly expressing MKI67, TOP2A, LYZ, NKG7, and DCN; 4) satellite cells specifically expressing the markers PAX7, MYF5, MYOD1, ASB5, and SOX8; 5) MSCs with high expression of PDGFRA, CXCL12, THY1, IGFBP7, and SFRP2; 6) T cells highly expressing CD2, CD3D, TRAC, and TRBC2; 7) osteoblasts characterized with high expression of ALPL, POSTN, COL1A1, IBSP, SPP1, MMP13, and RUNX2; 8) plasma cells expressing CD79A, JCHAIN, MZB1, and IGHG1; 9) chondrocytes specifically expressing ACAN, COL2A1, COL11A2, and COL9A1; 10) mononuclear phagocytes (MPs) expressing LYZ, CD14, AIF1, CD1C, FCER1A, MRC1, and C1QC; 11) mural cells specifically expressing RGS5, ACTA2, TAGLN, MYLK, and MYH11; 12) endothelial cells (ECs) highly expressing CDH5, PECAM1, VWF, and CLDN5; and 13) fibroblasts highly expressing DCN, COL1A2, and COL1A1. The proportion of main cell types in HOE tissues was compared with that in the control group, as shown in Figure 1C. We observed that almost all 13 types of cell populations were present in each sample, except for the osteoblasts, which were specifically identified in HOE sample A1, not in control sample B2. The heatmap of the top 10 differentially expressed gene sets of these 13 cell clusters is shown in Figure 1D. SP7 (OSX), RUNX2, and IHH are three key representative genes that control bone formation. The expression profiles of these three representative genes in the cell populations are shown in Figure 1E. We observed that SP7 was specifically expressed in osteoblasts, while RUNX2 was expressed in both osteoblasts and chondrocytes. IHH is an upstream regulator of RUNX2 and SP7 during osteoblast differentiation from MSCs. IHH is expressed in the early stage of the process, so IHH was barely detected in later osteoblasts, as shown in Figure 1E.

### Heterogeneity characterization of osteoblasts in heterotopic ossification of the elbow

**FIGURE 2:**
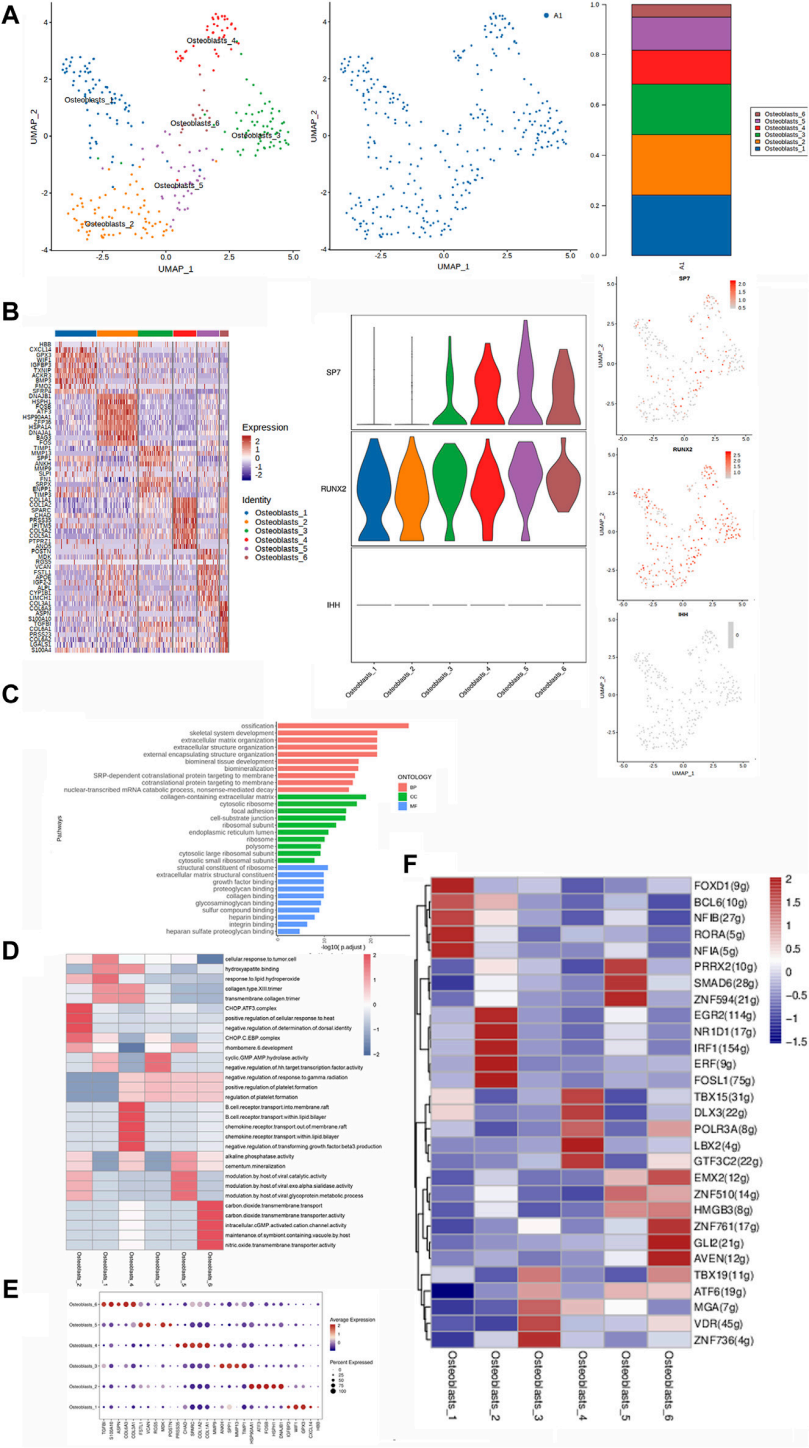
Heterogeneity characterization of osteoblasts in heterotopic ossification of the elbow. **(A)** UMAP analysis of osteoblasts showing six subclusters in HOE. The proportions of each subcluster are shown in the right panel. A1: HOE samples. **(B)** Heatmap of the top 10 differentially expressed gene sets of six subclusters. The right panel shows the expression profiles of three key representative genes SP7, RUNX2, and IHH in the cell subsets. **(C)** GO enrichment analysis shows upregulated pathways in osteoblasts of HOE. **(D)** Heatmap of the GSVA of the hallmark gene sets among the six osteoblast subclusters. **(E)** Dot plots showing the 30 signature gene expressions of the six subclusters. The size of dots represents the proportion of cells expressing a particular marker, and the spectrum of color refers to the mean expression levels of the markers. **(F)** Heatmap of the differentially expressed transcriptional factors of the six osteoblast subclusters.

We performed dimensionality reduction analysis of the osteoblast cluster. Unbiased clustering of the osteoblasts identified six subclusters in total based on UMAP analyses according to their gene profiles and canonical markers (Figure 2A). The proportion of six subclusters in HOE tissue A1 is shown in the right panel of Figure 2A.

The heatmap of the top 10 differentially expressed gene sets of these six osteoblast subclusters is shown in the left panel of Figure 2B. As demonstrated in the list of the top DEGs in Figure 2B, specifically, osteoblasts_1 expressed high levels of genes including HBB, CXCL14, GPX3, WIF1, IGFBP3, TXNIP, ACKR3, BMP3, FMO2, and SFRP4; osteoblasts_2 expressed high levels of genes including DNAJB1, HSPH1, FOSB, ATF3, HSP90AA1, ZFP36, HSPA1A, DNAJA1, BAG3, and FOS; osteoblasts_3 expressed high levels of genes including TIMP1, MMP13, SPP1, ANKH, MMP9, SLPI, FN1, SRPX, ENPP1, and TIMP3; osteoblasts_4 expressed high levels of genes including COL1A1, COL1A2, SPARC, CHAD, PRSS35, IFITM5, COL5A2, COL5A1, PTPRZ1, and ANO5; osteoblasts_5 expressed high levels of genes including POSTN, MDK, RGS5, VCAN, FSTL1, APOE, IGF2−2, ALPL, CYP1B1, and LIMCH1; and osteoblasts_6 expressed high levels of genes including COL3A1, COL6A3, ASPN, S100A10, TGFBI, COL6A1, PRSS23, COL6A2, LGALS1, and S100A4.

We then examined the three key representative genes SP7, RUNX2, and IHH that control bone formation. The expression profiles of these three representative genes in the cell subsets are given in the right panel of Figure 2B. SP7 was highly expressed in osteoblasts_3, osteoblasts_4, osteoblasts_5, and osteoblasts_6. RUNX2 was expressed in all six subclusters of osteoblasts. As an upstream gene of RUNX2 and SP7, IHH was barely detected in all six subclusters of osteoblasts. GO enrichment analysis (Figure 2C) indicated that osteoblasts in HOE were enriched in genes associated with pathways of ossification, skeletal system development, extracellular matrix organization, extracellular structure organization, biomineralization, cotranslational protein targeting to the membrane, etc.

The GSVA of the hallmark gene sets among the six osteoblast subclusters, as shown in Figure 2D, showed that osteoblasts_1 showed heightened activities of the collagen type XIII trimer, response to lipid hydroperoxide, and cyclic GMP.AMP hydrolase activity; osteoblasts_2 showed heightened activities of the CHOP.ATF3 complex, CHOP.C.EBP complex, and lipid hydroperoxide; osteoblasts_3 showed heightened activities of cyclic GMP.AMP hydrolase activity; osteoblasts_4 were enriched with hydroxyapatite binding and alkaline phosphatase; osteoblasts_5 were enriched with alkaline phosphatase activity; and osteoblasts_6 were enriched with intracellular cGMP-activated cation channels and alkaline phosphatase activities. The dot plots were used to compare the proportion of osteoblasts expressing subcluster-specific markers and their scaled relative expression levels, as shown in Figure 2E. Osteoblasts_1 showed higher expressions of WIF1 and GPX3. Osteoblasts_2 showed higher expression levels of HSP90AA1, ATF3, FOSB, HSPH1, and DNAJB1. Osteoblasts_3 showed higher expression levels of ANKH, SPP1, MMP13, and TIMP1. Osteoblasts_4 showed higher expression levels of PRSS35, CHAD, SPARC, COL1A2, and COL1A1. Osteoblasts_5 showed higher expression levels of FSTL1, VCAN, and MDK. Osteoblasts_6 showed higher expression levels of TGFB1, S100A10, COL6A3, and COL3A1. The heatmap of the differentially expressed transcriptional factors of the six osteoblast subclusters is shown in Figures 2F. Osteoblasts_1 showed higher expression levels of transcriptional factors FOXD1, RORA, NFIA, NFIB, and BCL6. Osteoblasts_2 showed higher expression levels of transcriptional factors EGR2, NR1D1, IRF1, ERF, and FOSL1. Osteoblasts_3 showed higher expression levels of transcriptional factors ZNF736, VDR, and MGA. Osteoblasts_4 showed higher expression levels of transcriptional factors LBX2, GTF3C2, and DLX3. Osteoblasts_5 showed higher expression levels of transcriptional factors ZNF594, SMAD6, and PRRX2. Osteoblasts_6 showed higher expression levels of transcriptional factors AVEN, GLI2, ZNF761, and EMX2.

### Heterogeneity characterization of chondrocytes in heterotopic ossification of the elbow

**FIGURE 3:**
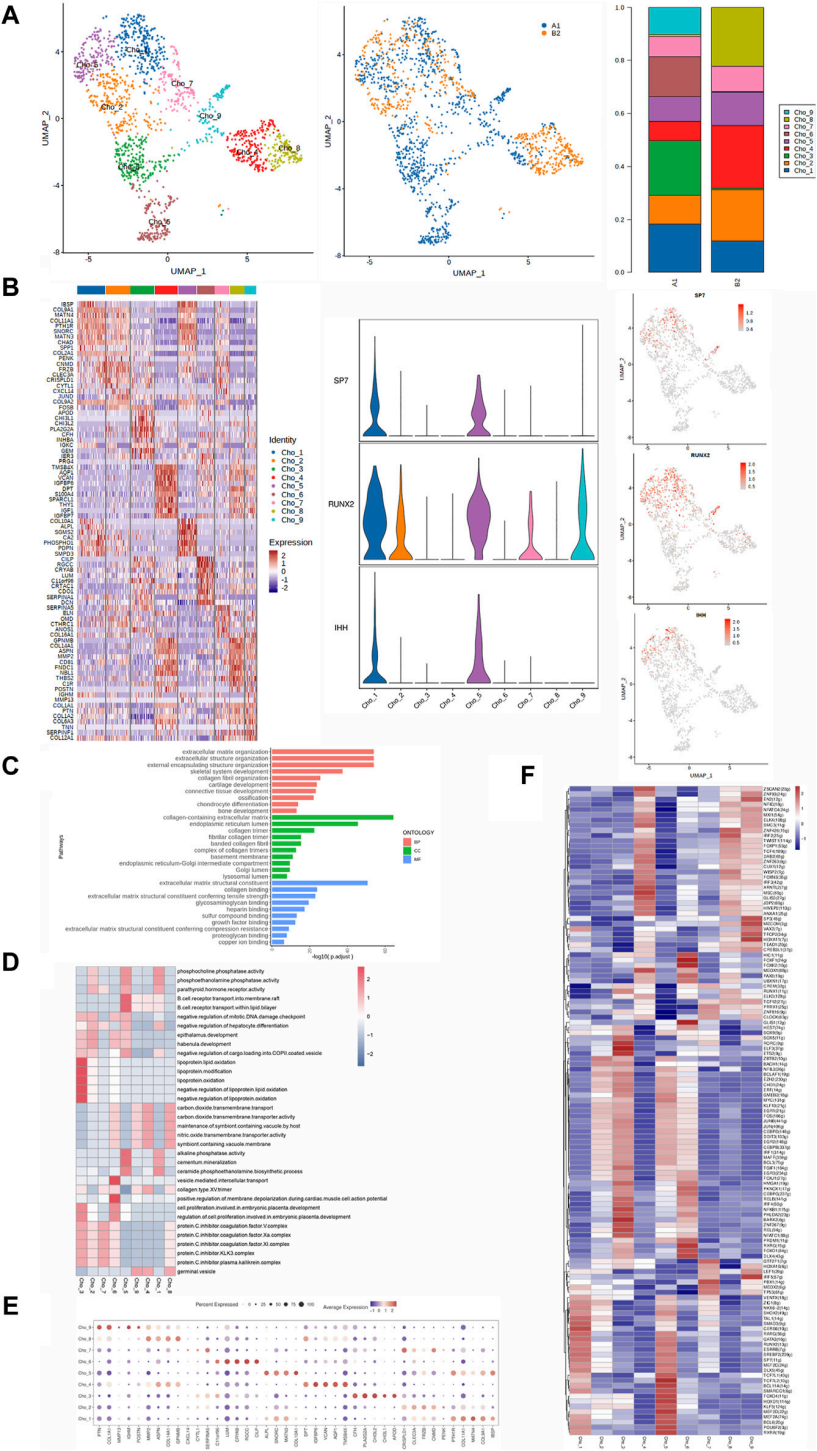
Heterogeneity characterization of chondrocytes in heterotopic ossification of the elbow. **(A)** UMAP analysis of chondrocytes showing nine subclusters in HOE. The proportions of each subcluster are given in the right panel. A1: HOE samples; B2: control samples. **(B)** Heatmap of the top 10 differentially expressed gene sets of nine subclusters. The right panel shows the expression profiles of three key representative genes SP7, RUNX2, and IHH in the cell subsets. **(C)** GO enrichment analysis showing upregulated pathways in chondrocytes of HOE. **(D)** Heatmap of the GSVA of the hallmark gene sets among the nine chondrocyte subclusters. **(E)** Dot plots showing the 30 signature gene expressions of the nine subclusters. The size of dots represents the proportion of cells expressing the particular marker, and the spectrum of color refers to the mean expression levels of the markers. **(F)** Heatmap of the differentially expressed transcriptional factors of the nine chondrocyte subclusters.

We performed dimensionality reduction analysis of the chondrocyte cluster. Unbiased clustering of the chondrocyte identified nine subclusters based on UMAP analyses according to their gene profiles and canonical markers (Figure 3A). The proportion of nine subclusters in HOE tissue A1 and control tissue B2 is shown in the right panel of Figure 3A.

The heatmap of the top 10 differentially expressed gene sets of these nine chondrocyte subclusters is shown in the left panel of Figure 2B. As shown in the list of the top DEGs in Figure 2B, chondrocyte_1 (Cho_1) expressed high levels of genes including IBSP, COL9A1, MATN4, COL11A1, PTH1R, SNORC, MATN3, CHADK, SPP1, and COL2A1. Chondrocyte_2 (Cho_2) expressed high levels of genes including PENK, CNMD, FRZB, CLEC3A, CRISPLD1, CYTL1, CXCL14, JUND, COL9A2, and FOSB. Chondrocyte_3 (Cho_3) expressed high levels of genes including APOD, CHI3L1, CHI3L2, PLA2G2A, CFH, INHBA, IGKC, GEM, IER3, and PRG4. Chondrocyte_4 (Cho_4) expressed high levels of genes including TMSB4X, AQP1, VCAN, IGFBP6, DPT, S100A4, SPACRL1, THY1, IGF1, and IGFBP7. Chondrocyte_5 (Cho_5) expressed high levels of genes including COL10A1, ALPL, SGMS2, CA2, PHOSPHO1, PDPN, and SMPD3. Chondrocyte_6 (Cho_6) expressed high levels of genes including CILP, RGCC, CRYAB, LUM, C11orf96, CRTAC1, CDO1, SERPINA1, and DCN. Chondrocyte_7 (Cho_7) expressed high levels of genes including SERPINA5, ELN, OMD, CTHRC1, ANOS1, and COL16A1. Chondrocyte_8 (Cho_8) expressed high levels of genes including GPNMB, COL14A1, ASPN, MMP2, CD81, FNDC1, NBL1, THBS2, C1R, and POSTN. Chondrocyte_9 (Cho_9) expressed high levels of genes including IGHM, MMP13, COL1A1, PTN, COL1A2, COL6A3, TNN, SERPINF1, and COL12A1.

We then examined the three key representative genes SP7, RUNX2, and IHH that control bone formation. The expression profiles of these three representative genes in the cell subsets are given in the right panel of Figure 3B. SP7 and IHH were detected in Cho_1 and Cho_5. RUNX2 was expressed in Cho_1, Cho_2, Cho_5, Cho_7, and Cho_9. GO enrichment analysis (Figure 3C) indicated that chondrocytes in HOE were enriched in genes associated with pathways of extracellular matrix organization, external encapsulation of the structural organization, skeletal system development, collagen fibril organization, cartilage development, ossification, chondrocyte differentiation, and bone development.

The GSVA of the hallmark gene sets among the nine chondrocyte subclusters, as shown in Figure 3D, showed that Cho_1 showed heightened activities of alkaline phosphatase, phosphocholine phosphatase, and the parathyroid hormone receptor; Cho_2 showed heightened parathyroid hormone receptor activity; Cho_3 showed heightened activities of cell proliferation and the protein C inhibitor KLK3 complex; Cho_4 was enriched with the collagen type XV trimer and alkaline phosphatase; Cho_5 was enriched with alkaline phosphatase activity and phosphocholine phosphatase activity; Cho_6 was enriched with cell proliferation and the collagen type XV trimer; Cho_7 was enriched with the protein C inhibitor KLK3 complex, collagen type XV trimer, and parathyroid hormone receptor activity; Cho_8 was enriched with the collagen type XV trimer activity and protein C inhibitor KLK3 complex; and Cho_9 was enriched with collagen type XV trimer activity.

The dot plots were used to compare the proportion of chondrocytes expressing subcluster-specific markers and their scaled relative expression levels, as shown in Figure 3E. Cho_1 showed higher expression levels of COL11A1, MATN4, COL9A1, and PTH1R. Cho_2 showed higher expression levels of CLEC3A, FAZB, and CNMD. Cho_3 showed higher expression levels of CFH, PLA2G2A, and CH3L2. Cho_4 showed higher expression levels of DPT, IGF8P6, VCAN, AQP1, and TMSB4X. Cho_5 showed higher expression levels of ALPL, SNOAC, MATN3, PTH1R, and IBSP. Cho_6 showed higher expression levels of LUM, CRYAB, RGCC, and CILP. Cho_7 showed higher expression levels of SERPINA5, CAISPLD1, and CNMD. Cho_8 showed higher expression levels of MMP2, ASPN, GPNMB, and COL14A1. Cho_9 showed higher expression levels of PTN, IGHM, and COL1A1. The heatmap of the differentially expressed transcriptional factors of the nine chondrocyte subclusters is shown in Figure 3F. Cho_1 showed higher expression levels of transcriptional factors CERS6, TAL1, SOX9, RUNX2, and SMAD3. Cho_2 showed higher expression levels of transcriptional factors ZBTB2, BACH1, ZIC1, and BCLAF1. Cho_3 showed higher expression levels of transcriptional factors SOX9, SOX5, RORC, ELF3, ETS2, BARX2, NFKB1, and REL. Cho_4 showed higher expression levels of transcriptional factors MEOX1, ELK3, MSC, SMC3, ZNF93, and ZSCAN2. Cho_5 showed higher expression levels of transcriptional factors TCF7L1, MEF2D, RXRA, POU6F2, RUNX2, and SOX9. Cho_6 showed higher expression levels of transcriptional factors RXRG, DLX4, PKNOX1, CEBPG, GLIS1, FOXF1, and FOXK2. Cho_7 showed higher expression levels of transcriptional factors PBX1, GTF1F1, ZNF816, and PRRX1. Cho_8 showed higher expression levels of transcriptional factors IRF2, ZNF426, WISP2, TCF12, and JDP2. Cho_9 showed higher expression levels of transcriptional factors IRF5, SP3, MECOM, HOXA11, MECOM, and EN2.

### Heterogeneity characterization of fibroblasts in heterotopic ossification of the elbow

**FIGURE 4:**
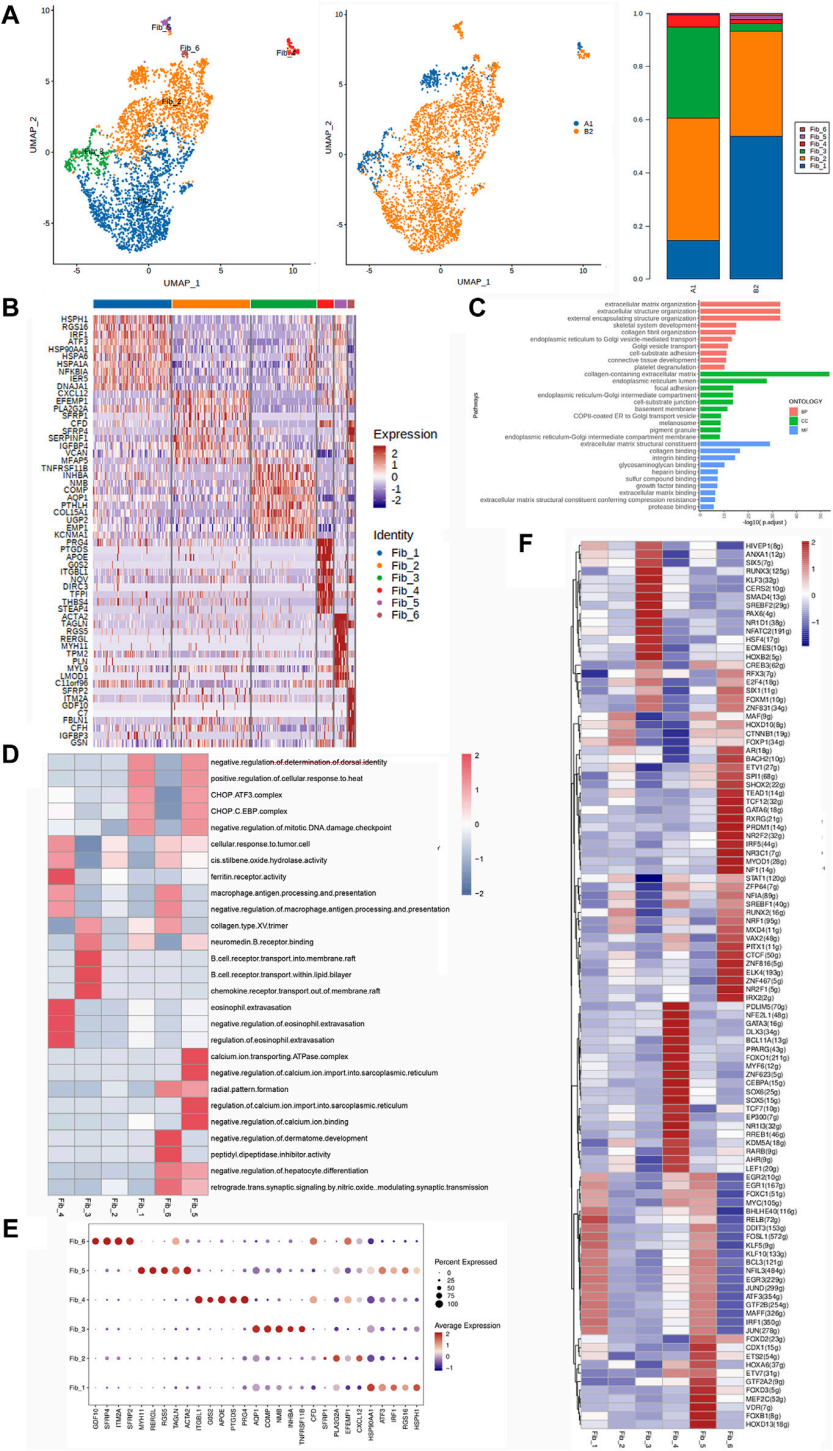
Heterogeneity characterization of fibroblasts in heterotopic ossification of the elbow. **(A)** UMAP analysis of fibroblast showing six subclusters in HOE. The proportions of each subcluster are given in the right panel. A1: HOE samples; B2: control samples. **(B)** Heatmap of the top 10 differentially expressed gene sets of six subclusters. **(C)** GO enrichment analysis showing upregulated pathways in fibroblasts of HOE. **(D)** Heatmap of the GSVA of the hallmark gene sets among the six fibroblast subclusters. **(E)** Dot plots showing the 30 signature gene expressions of the six subclusters. The size of dots represents the proportion of cells expressing the particular marker, and the spectrum of color refers to the mean expression levels of the markers. **(F)** Heatmap of the differentially expressed transcriptional factors of the six fibroblast subclusters.

We performed dimensionality reduction analysis of the fibroblast cluster. Unbiased clustering of the fibroblasts identified six subclusters in total based on UMAP analyses according to their gene profiles and canonical markers (Figure 4A). The proportion of the six subclusters in HOE tissue A1 and control tissue B2 is shown in the right panel of Figure 4A.

The heatmap of the top 10 differentially expressed gene sets of these six fibroblast subclusters is shown in Figure 4B. As shown in the list of the top DEGs in Figure 4B, specifically, fibroblasts_1 (Fib_1) expressed high levels of genes including HSPH1, RGS16, IRF1, ATF3, HSP90AA1, HSPA6, HSPA1A, NFKB1A, IER5, and DNAJA1; Fib_2 expressed high levels of genes including CXCL12, EFEMP1, PLA2G2A, SFRP1, CFD, SFRP4, SERPINF1, VCAN, and MFAP5; Fib_3 expressed high levels of genes including TNFRSF11B, INHBA, NMB, COMP, AQP1, PTHLH, COL15A1, UGP2, EMP1, and KCNMA1; Fib_4 expressed high levels of genes including PRG4, PTGDS, APOE, G0S2, ITGBL1, NOV, DIRC3, TFP1, THBS4, and STEAP4; Fib_5 expressed high levels of genes including ACTA2, TAGLN, RGS5, RERGL, MYH11, TPM2, PLN, MYL9, LMOD1, and C11orf96; and Fib_6 expressed high levels of genes including SFRP2, ITM2A, GDF10, C7, FBLN1, CFH, IGFBP3, and GSN. GO enrichment analysis (Figure 4C) indicated that fibroblasts in HOE were enriched in genes associated with pathways of extracellular matrix organization, extracellular structure organization, skeletal system development, collagen fibril organization, cell–substrate adhesion, connective tissue development, etc.

The GSVA of the hallmark gene sets among the six fibroblast subclusters, as shown in Figure 4D, showed that Fib_1 showed heightened activities of the CHOP.ATF3 complex, CHOP.C.EBP complex, and collagen type XIII trimer; Fib_2 showed heightened cis-stilbene oxide hydrolase activity; Fib_3 showed heightened activities of chemokine receptor transport and the collagen type XIII trimer; Fib_4 was enriched with activities of the ferritin receptor and macrophage antigen processing and presentation; Fib_5 was enriched with activities of the regulation of calcium import, calcium ion binding, CHOP.ATF3 complex, and CHOP.C.EBP complex; and Fib_6 was enriched with activities of macrophage antigen processing and presentation and collagen type XIII trimer. The dot plots were used to compare the proportion of fibroblasts expressing subcluster-specific markers and their scaled relative expression levels, as shown in Figure 4E. Fib_1 showed higher expression levels of HSP90AA1, HSPH1, and IRF1. Fib_2 showed higher expression levels of PLA2G2A and CXCL12. Fib_3 showed higher expression levels of AQP1, COMP, NMB, TNFRSF11B, and INHBA. Fib_4 showed higher expression levels of ITGBL1, APOE, PRG4, PTGDS, and G0S2. Fib_5 showed higher expression levels of MYH11, ACTA2, RGS5, RERGL, and TAGLN. Fib_6 showed higher expression levels of SFRP2, ITM2A, SFRP4, and GDF10. The heatmap of the differentially expressed transcriptional factors of the six fibroblast subclusters is shown in Figure 4F. Fib_1 showed higher expression levels of transcriptional factors RELB, KLF5, FOSL1, EGR3, JUN, and IRF1. Fib_2 showed higher expression levels of transcriptional factors FOXP1, MAF, CTNNB1, RUNX2, and HOXD10. Fib_3 showed higher expression levels of transcriptional factors HOXB2, EOMES, HSF4, NFATC2, NR1D1, PAX6, SREBF2, SMAD4, CERS2, KLF3, RUNX3, SIX5, and ANXA1. Fib_4 showed higher expression levels of transcriptional factors PDLIM5, NFE2L1, GATA3, DLX3, BCL11A, FOXO1, MYF6, ZNF623, CEBPA, SOX6, SOX5, TCF7, EP300, NR113, RREB1, KDM5A, RARB, AHR, and LEF1. Fib_5 showed higher expression levels of transcriptional factors HOXD13, FOXB1, VDR, MEF2C, FOXD3, GTF2A2, CDX1, ETS2, HOXA6, and KLF10. Fib_6 showed higher expression levels of transcriptional factors AR, BACH2, SPI1, SHOX2, TEAD1, TCF12, GATA6, RXRG, PRDM1, NR2F2, IRF5, NR3C1, MYOD1, NF1, RUNX2, MXD4, VAX2, PITX1, CTCF, ZNF816, ELK4, ZNF467, NR2F1, and IRX2.

### Heterogeneity characterization of MPs in heterotopic ossification of the elbow

**FIGURE 5:**
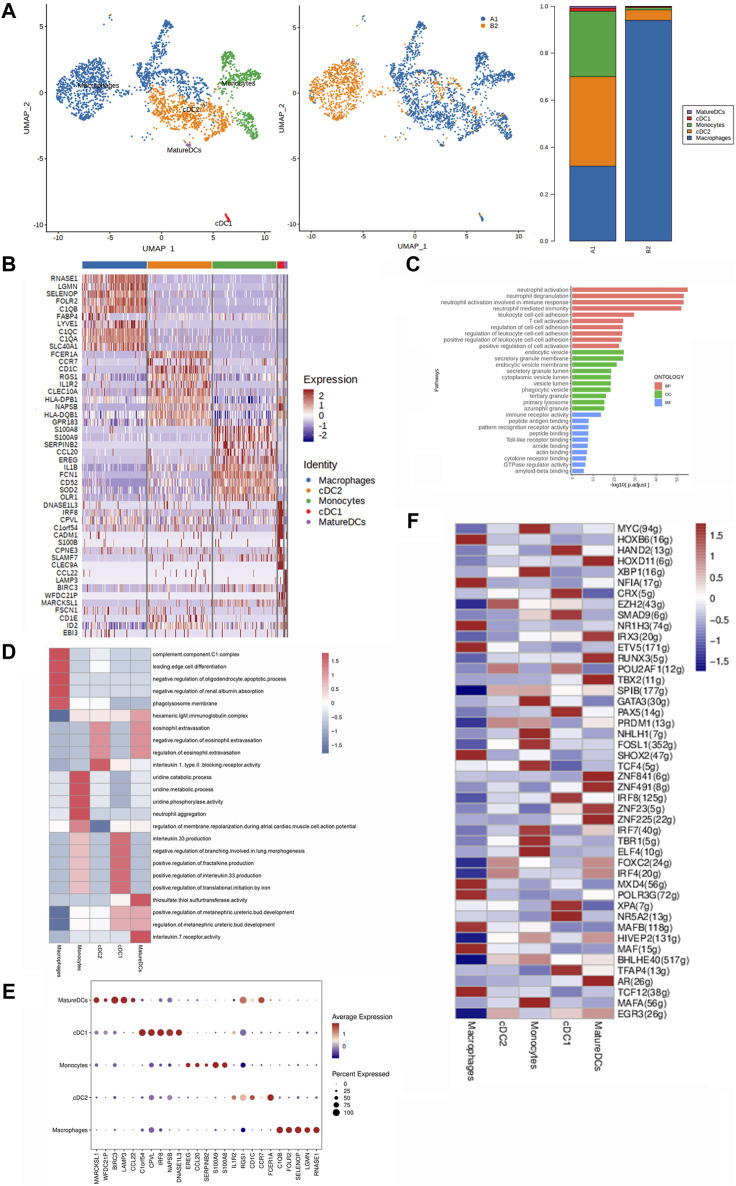
Heterogeneity characterization of MPs in heterotopic ossification of the elbow. **(A)** UMAP analysis of MPs showing five subclusters in HOE. The proportions of each subcluster are given in the right panel. A1: HOE samples; B2: control samples. **(B)** Heatmap of the top 10 differentially expressed gene sets of the five subclusters. **(C)** GO enrichment analysis showing upregulated pathways in MPs of HOE. **(D)** Heatmap of the GSVA of the hallmark gene sets among the five MP subclusters. **(E)** Dot plots showing the 30 signature gene expressions of the five subclusters. The size of dots represents the proportion of cells expressing the particular marker, and the spectrum of color refers to the mean expression levels of the markers. **(F)** Heatmap of the differentially expressed transcriptional factors of the five MP subclusters.

We performed dimensionality reduction analysis of the mononuclear phagocyte cluster. Unbiased clustering of the MPs identified five subclusters in total based on UMAP analyses according to their gene profiles and canonical markers (Figure 5A): mature DCs, cDC1, monocytes, cDC2, and macrophages. The proportion of the five subclusters in HOE tissue A1 and control tissue B2 is shown in the right panel of Figure 5A.

The heatmap of the top 10 differentially expressed gene sets of these 5 MP subclusters is shown in Figure 5B. Specifically, mature DCs expressed high levels of genes including RNASE1, LGMN, SELENOP, FOLR2, C1QB, FABP4, LYVE1, C1QC, C1QA, and SLC40A1; cDC1 expressed high levels of genes including FCER1A, CCR7, CD1C, RGS1, IL1R2, CLEC10A, HLA-DPB1, NAPSB, HLA-DQB1, and GPR183; monocytes expressed high levels of genes including S100A8, S100A9, SERPINB2, CCL20, EREG, IL1B, FCN1, CD52, SOD2, and OLR1; cDC2 expressed high levels of genes including DNASE1L3, IRF8, CPVL, C1orf54, CADM1, S100B, CPNE3, SLAMF7, and CLEC9A; and macrophages expressed high levels of genes including CCL22, LAMP3, BIRC3, WFDC21P, MARCKSL1, FSCN1, CD1E, ID2, and EBI3. GO enrichment analysis (Figure 5C) indicated that MPs in HOE were enriched in genes associated with pathways of neutrophil activation, neutrophil degranulation, T-cell activation, regulation of cell–cell adhesion, etc.

The GSVA of the hallmark gene sets among the five MP subclusters, as shown in Figure 5D, showed that mature DCs showed heightened activities of the IL-7 receptor, thiosulfate thiol sulfurtransferase, and eosinophil extravasation; cDC1 showed heightened activities of IL-33 production, fractalkine production, and translational initiation by iron; monocytes showed heightened activities of uridine phosphorylase and neutrophil aggregation; cDC2 was enriched with activities of the IL-1 receptor and eosinophil extravasation; and macrophages were enriched with activities of the complement component C1 complex, edge cell differentiation, oligodendrocyte apoptotic process, and phagolysosome membrane. The dot plots were used to compare the proportion of MPs expressing subcluster-specific markers and their scaled relative expression levels, as shown in Figure 5E. Mature DCs showed higher expression levels of BIRC3, LAMP2, RGS1, CCR7, and MARCKSL1. cDC1 showed higher expression levels of C1orf54, CPVL, IRF8, NAPSB, and DNASE1L3. Monocytes showed higher expression levels of EREG, S100A9, S100A8, and CCL20. cDC2 showed higher expression levels of FCER1A, CD1C, and IL1R2. Macrophages showed higher expression levels of C1QB, SELENOP, RNASE1, FOLR2, and LGMN. The heatmap of the differentially expressed transcriptional factors of the five MP subclusters is shown in Figure 5F. Mature DCs showed higher expression levels of transcriptional factors HOXD11, IRX3, RUNX3, TBX2, ZNF841, ZNF491, ZNF23, ZNF225, and AR. cDC1 showed higher expression levels of transcriptional factors HAND2, CRX, SMAD9, PAX5, IRF8, XPA, NR5A2, and TFAP4. Monocytes showed higher expression levels of transcriptional factors MYC, XBP1, GATA3, NHLH1, FOSL1, TCF4, IRF7, TBR1, ELF4, and MAFA. cDC2 showed higher expression levels of transcriptional factors EZH2, POU2AF1, PRDM1, FOXC2, IRF4, and EGR3. Macrophages showed higher expression levels of transcriptional factors HOXB6, NFIA, NR1H3, ETV5, SHOX2, MXD4, POLR3G, MAFB, MAF, and TCF12.

### Trajectory analysis of osteoblasts in HOE

**FIGURE 6:**
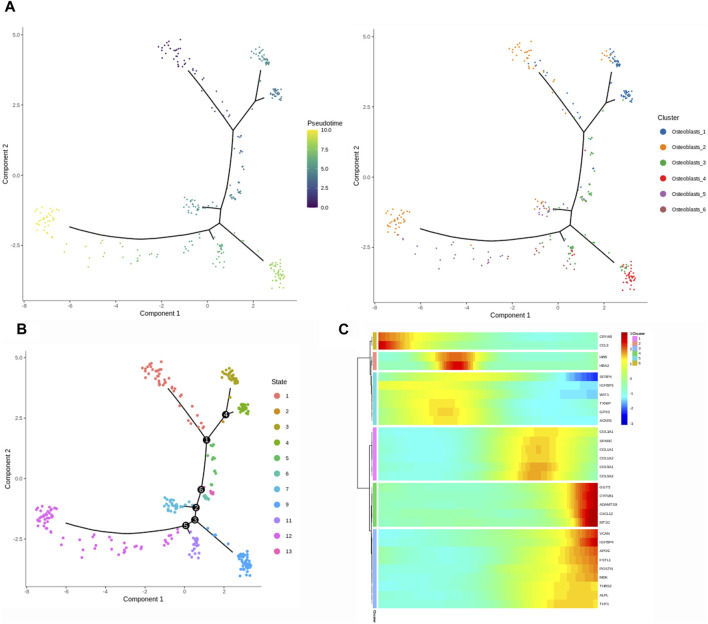
Trajectory analysis of osteoblasts in HOE. **(A)** The Monocle 2 trajectory plot shows the pseudotime curve and the dynamics of six osteoblast subclusters. **(B)** Distribution of distinct osteoblast subclusters in different states. **(C)** Heatmap of the signature genes that were differentially expressed along the pseudotime, as indicated in HOE.

We performed the trajectory analysis of the osteoblasts based on the Monocle 2 algorithm to infer the osteoblast maturation course in HOE (Figure 6). The pseudotime curve and the dynamics of six osteoblast subclusters are shown by the Monocle 2 trajectory plot given in Figure 6A. The distribution of distinct osteoblast subclusters in different states is shown in Figure 6B. The heatmap of the signature genes that were differentially expressed, along with the pseudotime, is shown in Figure 6C. We observed that some genes were gradually downregulated along the trajectory differentiation process, such as CRYAB, CCL3, SFRP4, WIF1, and IGFBP3. Conversely, some other critical factors such as VCAN, IGFBP4, FSTL1, POSTN, MDK, THBS2, and ALPL were upregulated in the process.

### Cell–cell communication networks in HOE

**FIGURE 7:**
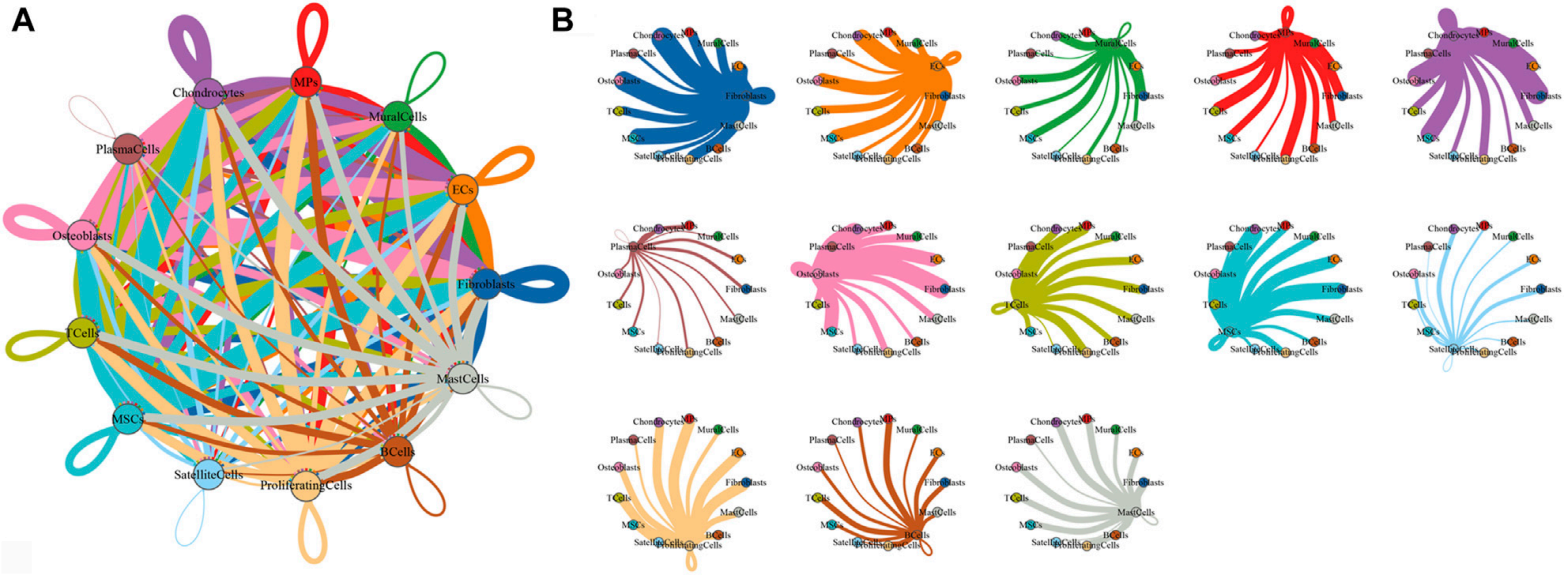
Analysis of the cell–cell interaction in HOE. **(A)** Network diagram of the cell–cell interaction of the 13 identified cell types in HOE. Each line color indicates the ligands expressed by the identified cell cluster represented in the same color. The lines connect to the cell clusters with the cognate receptors. The line thickness is proportional to the number of ligands related to cognate receptors. **(B)** Detailed view of the ligands expressed by each identified cell type and the cells with the cognate receptors.

**FIGURE 8:**
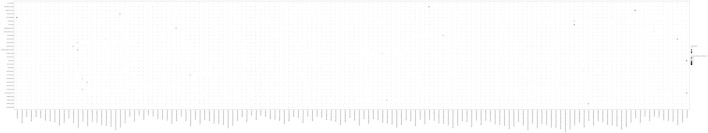
Dot plots showing the 30 signature ligand–receptor pairs across the 13 identified cell clusters. The size of dots represents *p*-values with the scale to the right, and the spectrum of color indicates the mean expression levels of interacting molecule 1 in cluster 1 and interacting molecule 2 in cluster 2.

To characterize the microenvironment of HOE tissues, CellPhoneDB was used to detect the intercellular communication among the 13 identified clusters in HOE. The analysis of ligand–receptor pairs demonstrated extensive molecular interactions among the 13 identified clusters in HOE, as shown in Figures 7A, B. Figure 8 shows the dot plots showing the 30 signature ligand–receptor pairs across the 13 identified cell clusters. Some ligand–receptor pairs were identified in this study. For example, the IL-6–HRH1 pair interaction was significant, as indicated by the big dot and in red in Figure 8, suggesting that IL-6 might be involved in the progression of HOE. Ligand pairs for COL24A1, COL22A1, VWF, FZD6, FGF2, and NOTCH1 were also identified, suggesting that multiple pathways may be responsible for HOE progression.

## Discussion

Heterotopic ossification is a disease with pathologic new formation in soft tissues. Current poor understanding of the underlying mechanisms of HO progression hampers the development of new potential treatment strategies.

Some progress has been made in HO studies. It has been reported that brain injuries tend to cause generalized heterotopic ossification, including in the hip, knee, and elbow or shoulder joints (Garland, 1988). Among hereditary HO, progressive osseous heteroplasia (POH) and Albright’s hereditary osteodystrophy (AHO) are considered to be intramembranous ossification, while fibrodysplasia ossificans progressiva (FOP) is considered to be endochondral ossification (Kaplan and Shore, 2000). The difference may be associated with their different pathogenesis. Trauma-induced HO might occur through endochondral osteogenesis (Wong et al., 2020). Trauma can result in elevated inflammatory cytokines such as TNFα, IL-1β, IL-6, and MCP-1, which could lead to abnormal activation of mesenchymal stem cells in the soft tissues (Sung Hsieh et al., 2017). Inflammation-associated cells, such as macrophages and mast cells, also accumulate at the site of trauma-induced HO and promote HO (Convente et al., 2018). It is reported that lymphoid tissues contribute to the cellular niche in HO (Loder et al., 2016). Our recent studies have indicated that inflammatory factors TNF-α and IL-6 are involved in thoracic ossification of the ligamentum flavum (Zhang et al., 2017; Huang et al., 2022).

It has been reported that some cell types are associated with HO. Increased mast cells have been documented in the cases of non-genetic HO, with mast cells showing near sites of ectopic bone formation on biopsies of HO at various sites (Convente et al., 2015). Lymphocytic inflammation has been demonstrated as a common histologic feature of HO (Kan et al., 2009), in particular perivascular lymphocytic inflammation as a consistent feature in peri-articular, non-genetic HO. In HO associated with cardiac valves, a polyclonal chronic inflammatory infiltrate is a common finding, including lymphocytes, mast cells, and plasma cells (Mohler et al., 2001; Steiner et al., 2007). Indeed, the cellular origin of HO is relatively complicated, and a variety of cells may have the potential to shift to osteogenic differentiation in response to some specific stimulus, which promotes HO formation. The traditional research approach is based on the mixture of cellular populations, which is unable to obtain sufficient resolution in the identification of specific cellular types to determine the heterogeneity in HO. Single-cell RNA sequencing has become a promising new approach to explore the heterogeneity of different diseases. The elbow is the first place for the development of heterotopic ossification among all joints; however, the cellular composition, dynamics, and characteristics of HOE are largely unknown. The aim of this study is to explore the cellular origin and progression of HOE by single-cell RNA sequencing analysis.

In this study, we identified thirteen clusters of cells in HOE and further analyzed the subclusters for four of the main cell types. The thirteen clusters were mast cells, B cells, proliferating cells, satellite cells, MSCs, T cells, osteoblasts, plasma cells, chondrocytes, MPs, mural cells, ECs, and fibroblasts. Moreover, we identified and analyzed six subclusters of osteoblasts, nine subclusters of chondrocytes, six subclusters of fibroblasts, and five subclusters of MPs. Figure 2C shows that HOE osteoblasts were enriched in genes associated with pathways of ossification, skeletal system development, extracellular matrix organization, extracellular structure organization, biomineralization, cotranslational protein targeting to the membrane, etc. These results suggest that cellular heterogeneity and these signaling pathways may contribute to the progression of HOE.

Figure 1E shows that OSX (SP7) was specifically expressed in osteoblasts, indicating that osteoblasts were characterized by OSX (SP7). The expression profiles in the osteoblast subclusters given in Figure 2B showed that SP7 was highly expressed in osteoblasts_3, osteoblasts_4, osteoblasts_5, and osteoblasts_6, suggesting that these subclusters may be responsible for OSX (SP7) to control osteoblast differentiation from stem cells during HOE progression. The heatmap of the differentially expressed transcriptional factors of the nine chondrocyte subclusters is shown in Figure 3F. Our observations showed that the key transcriptional factor for chondrocyte differentiation of SOX9 was expressed in chondrocyte subclusters Cho_1, Cho_3, and Cho_5, suggesting that these subclusters may be responsible for SOX9 to regulate chondrocyte differentiation from stem cells during HOE progression. These findings indicate that heterotopic ossification of the elbow is more likely mediated through endochondral ossification; however, we cannot rule out the possibility that intramembranous ossification is also involved in the progression of heterotopic ossification of the elbow.

Either endochondral ossification or intramembranous ossification will eventually lead to osteoblast differentiation and maturation. OSX (SP7) is an osteoblast-specific transcriptional factor that controls bone formation and osteoblast differentiation (Nakashima et al., 2002; Zhang et al., 2008). There is no bone formation without OSX. Interestingly, our results given in Figure 2B demonstrated that OSX was highly expressed in osteoblasts_3, osteoblasts_4, osteoblasts_5, and osteoblasts_6, not in subclusters osteoblasts_1 and osteoblasts_2, suggesting that subclusters of osteoblasts_3, osteoblasts_4, osteoblasts_5, and osteoblasts_6 are relatively more mature during osteoblastic progression of HOE. Distinct clusters and subclusters for various types of cells in HOE were identified with their corresponding signature gene sets. The trajectory analysis of the osteoblasts based on the Monocle 2 algorithm can infer the osteoblast maturation course in HOE, as shown in Figure 6. The pseudotime curve and the dynamics of six osteoblast subclusters were obtained. The heatmap of the signature genes that were differentially expressed with the pseudotime, as shown in Figure 6C, showed that some genes were gradually downregulated, such as CRYAB, CCL3, SFRP4, WIF1, and IGFBP3, while some other critical genes were upregulated along the trajectory differentiation process such as VCAN, IGFBP4, FSTL1, POSTN, MDK, THBS2, and ALPL, suggesting that these factors may participate in the progression of HOE. Furthermore, we addressed cell–cell communication networks in HOE in Figures 7, 8. The analysis of ligand–receptor pairs revealed extensive molecular interactions among the 13 identified clusters in HOE. Ligand pairs for IL-6, COL24A1, COL22A1, VWF, FZD6, FGF2, and NOTCH1 were identified, suggesting that multiple signaling pathways may be involved in HOE progression. Their roles and possible interactions with the osteoblast-specific master gene OSX (SP7) deserve further investigation. For example, the IL-6–HRH1 pair interaction was significant, as indicated by the big dot and in red in Figure 8, suggesting that IL-6 might participate in HOE. Our recent study has demonstrated that IL-6 is involved in the thoracic ossification of the ligamentum flavum (Huang et al., 2022). IL-6 activated osteoblastic gene expression such as BMP2, RUNX2, and OSX, indicating that IL-6 may favor heterotopic ossification.

In conclusion, this study provided the cellular atlas for HOE tissues by single-cell RNA sequencing analysis. We have established a greater extent of the heterogeneity of HOE cells than previously known through transcriptomic analysis at the single-cell level. We have observed gradual patterns of signature gene expression during the differentiation and maturation progression of osteoblasts in HOE with higher resolution. Further investigation of the heterogeneity of HOE cells may pave a way for the identification of potential targets for early diagnosis and therapeutic treatment of HOE.

## Data Availability

The raw data supporting the conclusion of this article will be made available by the authors, without undue reservation.
